# A Poxvirus Decapping Enzyme Colocalizes with Mitochondria To Regulate RNA Metabolism and Translation and Promote Viral Replication

**DOI:** 10.1128/mbio.00300-22

**Published:** 2022-04-18

**Authors:** Shuai Cao, Joshua A. Molina, Fernando Cantu, Candy Hernandez, Zhilong Yang

**Affiliations:** a Department of Veterinary Pathobiology, College of Veterinary Medicine & Biomedical Sciences, Texas A&M University, College Station, Texas, USA; b Division of Biology, Kansas State University, Manhattan, Kansas, USA; University of North Carolina, Chapel Hill

**Keywords:** decapping enzyme, poxvirus, vaccinia virus, mitochondria, translation, RNA decay, D10, translational control

## Abstract

Decapping enzymes remove the 5′ cap of eukaryotic mRNA, leading to accelerated RNA decay. They are critical in regulating RNA homeostasis and play essential roles in many cellular and life processes. They are encoded in many organisms and viruses, including vaccinia virus, which was used as the vaccine to eradicate smallpox. Vaccinia virus encodes two decapping enzymes, D9 and D10, that are necessary for efficient viral replication and pathogenesis. However, the underlying molecular mechanisms regulating vaccinia decapping enzymes’ functions are still largely elusive. Here, we demonstrated that vaccinia D10 almost exclusively colocalized with mitochondria. As mitochondria are highly mobile cellular organelles, colocalization of D10 with mitochondria can concentrate D10 locally and mobilize it to efficiently decap mRNAs. Mitochondria were barely observed in “viral factories,” where viral transcripts are produced, suggesting that mitochondrial colocalization provides a spatial mechanism to preferentially decap cellular mRNAs over viral mRNAs. We identified three amino acids at the N terminus of D10 that are required for D10’s mitochondrial colocalization. Loss of mitochondrial colocalization significantly impaired viral replication, reduced D10’s ability to remove the RNA 5′ cap during infection, and diminished D10’s gene expression shutoff and mRNA translation promotion abilities.

## INTRODUCTION

The methyl guanosine cap (m^7^G) at the 5′ end of eukaryotic mRNA regulates many aspects of RNA processing and metabolism, such as splicing, transportation to the cytoplasm, protecting mRNA from 5′ to 3′ degradation by exonucleases, and recruiting cap-dependent translation initiation factors (1). Decapping enzymes are proteins comprising many known members that regulate mRNA stability through removing the 5′ cap to render RNA sensitive to exonuclease-mediated 5′ to 3′ digestion. They are critical for regulating the homeostasis of cellular mRNAs levels and play crucial roles in numerous cellular and life processes (2). In humans and various model systems, decapping enzymes are involved in cell migration, development, and carcinogenesis (3–7). The enzymatic activity of decapping enzymes lies in the Nudix motif that hydrolyzes nucleoside diphosphate linked to other moieties (8). The Nudix motifs are highly conserved and often located in the center regions of the decapping enzymes (8). Positive and negative regulatory domains are typically presented at the N and C termini of the proteins, which bind to either RNAs or other proteins to regulate the substrate specificities and potentials of decapping enzymes (9–12), although the regulatory nature and mechanisms are largely unknown.

Dcp2 was the first discovered decapping enzyme from the budding yeast Saccharomyces cerevisiae (13), followed by numerous homologs found in other organisms, including humans and plants (4, 14–17). The human genome encodes multiple decapping enzymes (18). Dcp2 carries out its catalytic activity in cytoplasmic structures called processing bodies (P-bodies) (15). P-bodies are cytoplasmic ribonucleoprotein (RNP) granules containing proteins involved in RNA degradation, including decapping enzymes, exonuclease Xrn1, and proteins involved in the RNA interference pathway beside translationally repressed mRNAs (19). Local concentrating Dcp2 and other related proteins in P-bodies increase RNA degradation efficiency and translational repression (20); however, different decapping enzymes likely have distinct substrate specificities and modes of action. For example, Nudt16 is a nuclear decapping enzyme with a high affinity for U8 small nucleolar RNA (21), and Nudt12 is a cytoplasmic decapping enzyme that targets NAD^+^-capped RNA (22). While much progress has been made to understand decapping enzymes, how they achieve different functions and their molecular mechanisms of action remain largely elusive.

Notably, many viruses encode decapping enzymes; these include poxviruses, Africa swine fever virus, and many other large nucleocytoplasmic DNA viruses (23–26). Vaccinia virus (VACV), the vaccine used to eradicate historically one of the most (if not the most) devastating infectious diseases, smallpox, encodes two decapping enzymes, D9 and D10 (24, 25). Poxviruses are a large family of double-stranded DNA viruses causing many severe diseases in humans and economically and ecologically important animals (27, 28). They are also actively developed for treating cancers and as vaccine vectors (28). D10 is present in all sequenced poxviruses, including VACV (25). D9 and D10’s decapping activities were demonstrated *in vitro* (24, 25). They negatively regulate viral and cellular gene expression in VACV-infected cells by accelerating mRNA turnover, which is thought to be critical to controlling the VACV cascade gene expression program to ensure sharp transitions between different stages of viral replication (29–33). However, it is unclear if these viral decapping enzymes employ mechanisms to preferentially target cellular mRNAs in VACV-infected cells. VACV infection produces excessive RNAs, and some of them can form double-stranded RNA (dsRNA) to stimulate receptor 2′5′‐oligoadenylate synthetase 2 (OAS)-RNase L pathway and PKR activation, which lead to RNA decay and mRNA translation repression, respectively. D9 and D10 are among the essential viral factors to resolve excessive double-stranded RNA (dsRNA) produced in VACV infection to evade these antiviral responses (34). Our recent data identified another function of D9 and D10; they are required for efficient VACV mRNA translation during infection (35). Strikingly, D10 alone promotes viral mRNA translation in uninfected cells to ensure high levels of viral protein production to compensate for the decapping activity-induced RNA reduction (35). Promoting mRNA translation by D10 is unusual, as decapping enzymes are thought to negatively regulate RNA translation by competing with cap-binding translation initiation factors (19, 36–39). However, the mechanism by which D10 promotes mRNA translation is still largely unknown.

Here, we demonstrated that D10 colocalized with mitochondria almost exclusively, which is the first one discovered among known decapping enzymes. We further identified the amino acids at the N terminus of D10 that are required for D10's mitochondrial colocalization. The mitochondrial colocalization of D10 is required for efficient viral replication and D10’s ability to regulate mRNA metabolism and translation promotion. The results indicate that mitochondrial association provides D10 a spatial mechanism to concentrate proteins locally with remarkable mobility to preferentially decap cellular mRNAs during VACV infection.

## RESULTS

### VACV D10 colocalizes with mitochondria.

**FIG 1:**
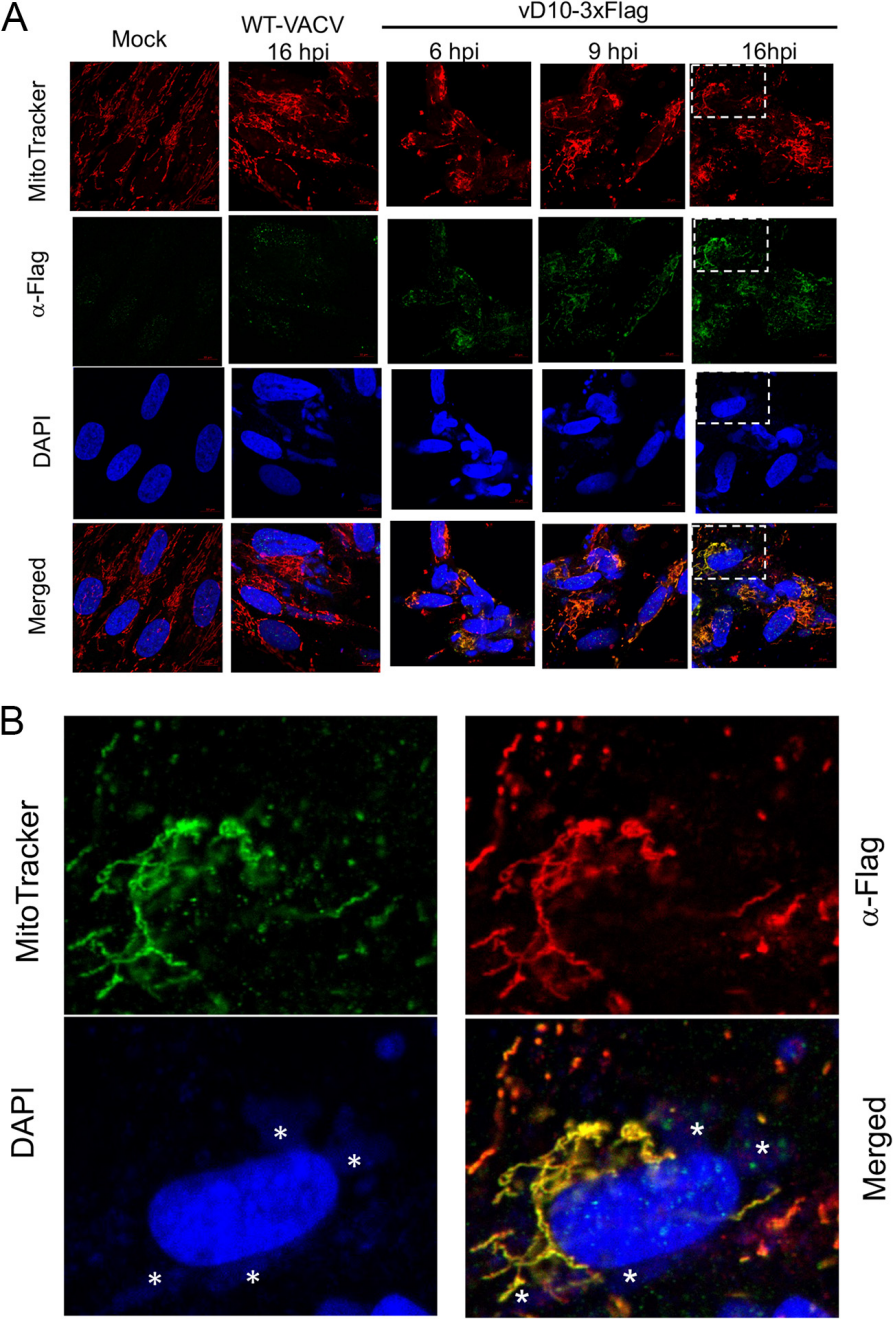

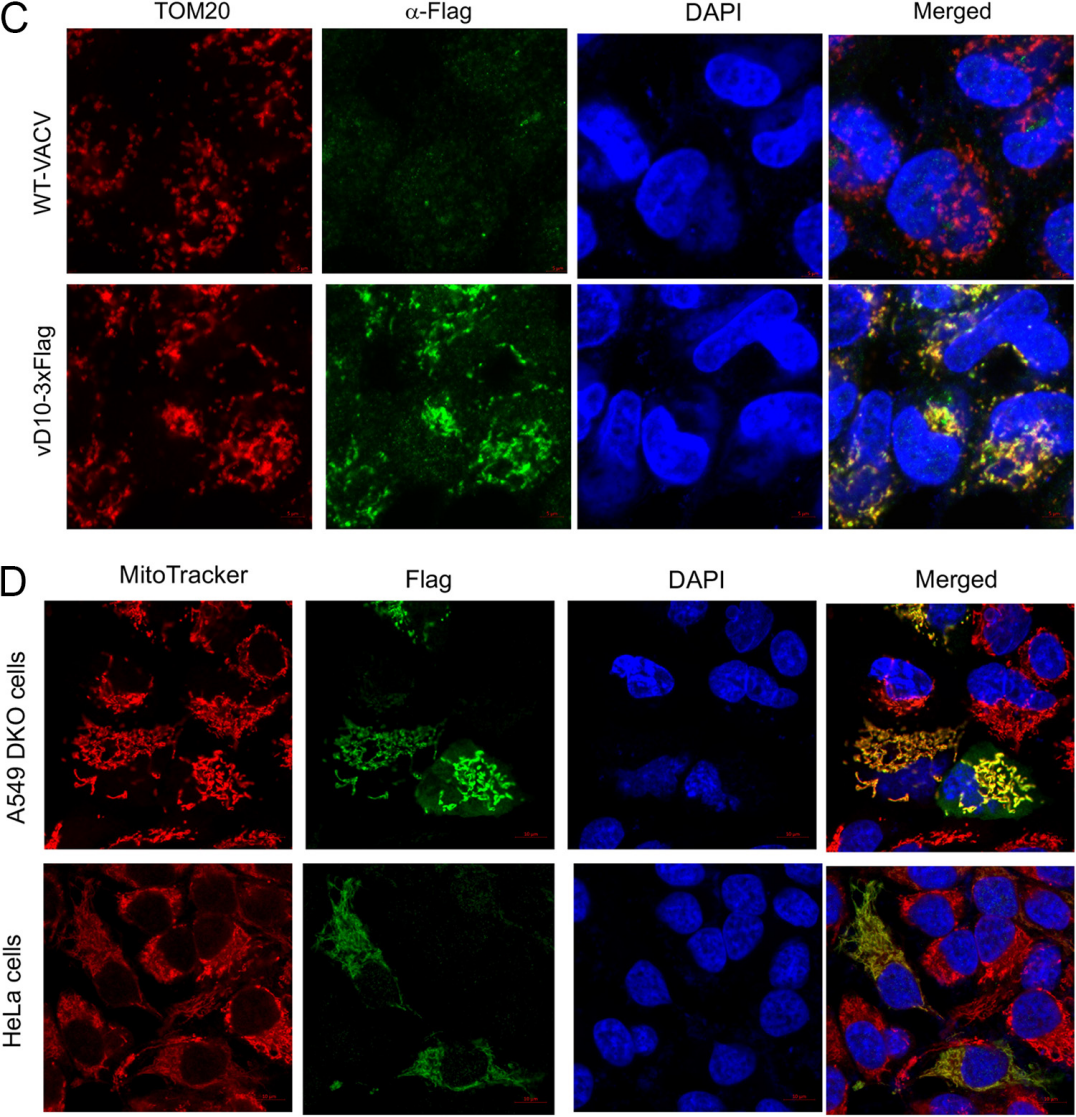
VACV D10 colocalizes with mitochondria. D10 with an N-terminal 3×Flag tag was expressed from VACV during infection or a plasmid in the absence of viral infection. Under each circumstance (with or without viral infection), colocalization of D10 with mitochondria was observed in more than one cell line with more than five independent experiments. Each picture shown in the figures is representative of six different views of that particular experiment with more than 100 cells in total. In all of the cells with D10 detected (by α-Flag antibody staining), D10 colocalized with mitochondria. (A) D10 colocalizes with mitochondria in HFFs during VACV infection. HFFs were infected with vD10-3xFlag or WT-VACV (MOI = 3), or mock infected. Confocal microscopy was used to visualize D10 (α-Flag antibody, green), mitochondria (MitoTracker, red), and DNA (DAPI, blue) at 6, 9, and 16 hpi. (B) Zoomed-in view of the indicated areas in panel A. Asterisks indicate viral factories. (C) D10 colocalizes with mitochondria in A549DKO cells during VACV infection. A549DKO cells were infected with vD10-3xFlag or WT-VACV (MOI = 3). Confocal microscopy was employed to visualize D10 (anti-Flag antibodies, green), mitochondria (α-Tom20 antibody, red), and DNA (DAPI, blue) at 16 hpi. (D) D10 colocalizes with mitochondria in uninfected cells. A549DKO or HeLa cells were transfected with plasmid encoding codon-optimized D10 with a C-terminal 3×Flag tag. Confocal microscopy was used to visualize D10 (α-Flag antibody, green), mitochondria (MitoTracker, red), and DNA (DAPI, blue) at 24 h posttransfection.

We examined D10’s subcellular localization using a recombinant VACV vD10-3xFlag in which D10 was tagged with a 3×Flag epitope at the C terminus. We used primary human foreskin fibroblasts (HFFs) and HeLa cells and found that D10 almost exclusively colocalized with mitochondria (Fig. 1A and B; Fig. S1 in the supplemental material). A549DKO human lung carcinoma cells in which the PKR and RNase L genes were knocked out via CRISPR/Cas9 are very useful in studying VACV decapping enzyme functions since it excludes the PKR and RNase L activation-related RNA degradation and translation repression during decapping enzyme-inactivated VACV infection (34). Again, D10 colocalized with mitochondria during infection, evidenced by its colocalization with MitoTracker or Tom20 in A549DKO cells. The latter is a well-known mitochondrial protein (40) (Fig. 1C; Fig. S2). Another notable observation is that mitochondria barely reside in the viral factories that are the cytoplasmic sites of viral replication with intensive viral DNA staining (Fig. 1A to C). Using a plasmid expressing D10 with a C-terminal 3×Flag, we observed D10 colocalized with mitochondria in uninfected A549DKO and HeLa cells (Fig. 1D). Together, our results demonstrated VACV D10’s colocalization with mitochondria either during viral infection or in uninfected cells.

10.1128/mbio.00300-22.1 FIG S1 D10 colocalizes with mitochondria in HeLa cells during infection. HeLa cells were infected with vD10-3xFlag, WT-VACV, or mock infected. Confocal microscopy was used to visualize D10 (α-Flag antibody, green), mitochondria (MitoTracker, red), and DNA (DAPI, blue) at 6, 9, and 16 hpi. Download FIG S1, TIF file, 2.9 MB. Copyright © 2022 Cao et al. 2022 Cao et al. https://creativecommons.org/licenses/by/4.0/ This content is distributed under the terms of the Creative Commons Attribution 4.0 International license.

10.1128/mbio.00300-22.2 FIG S2 D10 colocalizes with mitochondria in A549DKO cells during infection. A549DKO cells were infected with vD10-3xFlag or WT-VACV. Confocal microscopy was employed to visualize D10 (α-Flag antibody, green), mitochondria (MitoTracker, red), and DNA (DAPI, blue) at 16 hpi. Download FIG S2, TIF file, 1.2 MB. Copyright © 2022 Cao et al. 2022 Cao et al. https://creativecommons.org/licenses/by/4.0/ This content is distributed under the terms of the Creative Commons Attribution 4.0 International license.

### Identification of N-terminal hydrophobic amino acids required for D10 mitochondrial colocalization.

**FIG 2:**
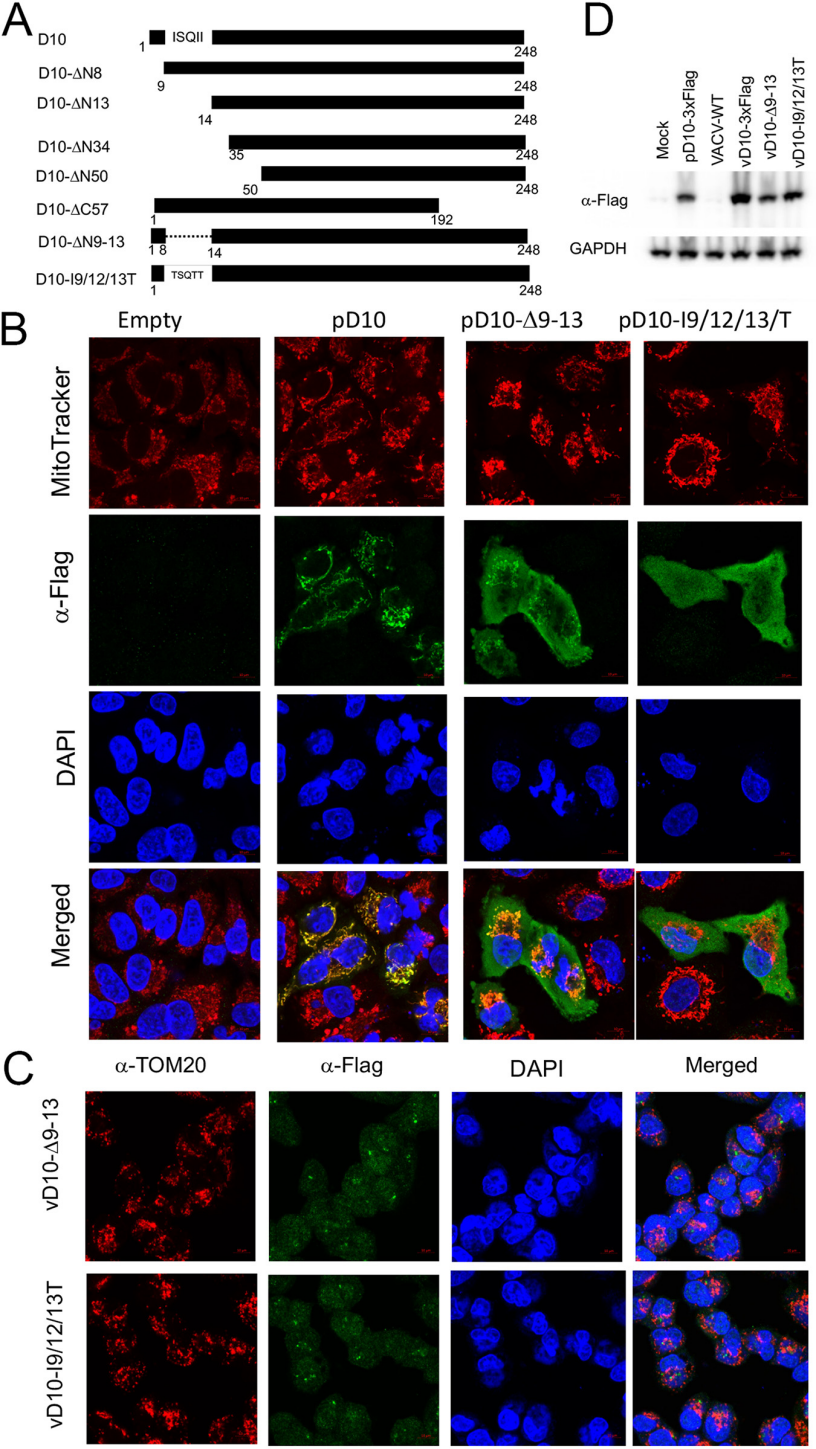
Three isoleucines located at the N-terminal hydrophobic region are required for D10 mitochondrial colocalization. (A) Schematic of D10 mutants used in this study. (B) A549DKO cells were transfected with a plasmid expressing indicated codon-optimized D10 truncation mutants with a C-terminal 3×Flag. Confocal microscopy was employed to visualize D10 or its mutants using α-Flag antibody (green), mitochondria (MitoTracker, red), and DNA (DAPI, blue) at 24 h posttransfection. (C) D10 with amino acids 9 to 13 mutation or deletion expressed from recombinant VACV does not colocalize with mitochondria during infection, A549DKO cells were infected with indicated recombinant VACVs (MOI = 3) encoding D10 mutants with a C-terminal 3×Flag tag. Confocal microscopy was used to visualize D10 (α-Flag antibody, green), mitochondria (α-Tom20, red), and DNA (DAPI, blue) at 16 hpi. For panels B and C, the importance of the three isoleucines at positions 9, 12, and 13 to mitochondrial colocalization was observed with at least three independent experiments using D10 mutant expressed from a plasmid or a recombinant VACV. Three to six views were pictured under each condition (plasmid or recombinant virus) with more than 50 cells pictured. In all the cells with D10 mutant expression detected (by α-Flag antibody staining), the loss of D10 colocalization with mitochondria was consistently observed. (D) The levels of D10 or its mutants from the recombinant VACVs are expressed at comparable levels. A549DKO cells were infected with indicated viruses (MOI = 3) or mock infected. Western blotting was employed to examine 3×Flag-tagged D10 expression using an α-Flag antibody.

We first generated and tested plasmids expressing D10 C-terminal (D10-ΔC57) and D10 N-terminal (D10-ΔN50) truncation mutants, respectively (Fig. 2A). While D10-ΔC57 remained to colocalize with mitochondria, the D10-ΔN50 lost its mitochondrial colocalization (Fig. 2B), suggesting that the N-terminal amino acids are required for D10 mitochondrial colocalization. Further truncations of D10 indicated that the N-terminal amino acids from 9 to 13 are needed for D10 colocalization with mitochondria because deletion of the first eight N-terminal amino acids did not fully block D10 mitochondria localization. In contrast, deletion of the first N-terminal 13 amino acids rendered D10 to lose its mitochondrial colocalization (Fig. 2A; Fig. S3). We further tested two additional D10 mutants, D10-Δ9-13, in which the amino acids from 9 to 13 (ISQII) were deleted, and D10-I9/12/13T, in which the hydrophobic isoleucine (I) at 9, 12, and 13 was changed to threonine (T, neutral) (Fig. 2A). The rationale of the latter is that those hydrophobic residues may be critical to forming a helix to interact with mitochondrial proteins and dock D10 on mitochondria. The deletion mutant (D10-Δ9-13) largely, while the point mutation mutant (D10-I9/12/13T) entirely, rendered D10 to lose its mitochondrial colocalization in uninfected cells (Fig. 2B).

10.1128/mbio.00300-22.3 FIG S3 D10 mitochondrial colocalization signal is located at the N terminus of D10. A549DKO cells were transfected with a plasmid expressing indicated codon-optimized D10 truncation mutants with C-terminal 3×Flag. Confocal microscopy was employed to visualize D10 or its mutants using α-Flag antibody (green). Download FIG S3, TIF file, 1.1 MB. Copyright © 2022 Cao et al. 2022 Cao et al. https://creativecommons.org/licenses/by/4.0/ This content is distributed under the terms of the Creative Commons Attribution 4.0 International license.

We then constructed two recombinant VACVs, vD10-I9/12/13T and vD10-Δ9-13, in which the D10 amino acids from 9 to 13 (ISQII) were mutated to TSQTT or deleted, respectively, yet both contained a 3×Flag tag at the C terminus. Interestingly, in both cases, the mutated D10 diffused in the infected A549DKO or HeLa cells but did not colocalize with mitochondria, using Tom20 or MitoTracker to locate mitochondria (Fig. 2C; Fig. S4 and S5). In addition, Western blotting showed comparable protein levels of D10 and its mutants produced from the recombinant viruses (Fig. 2D). These results corroborate that the N-terminal amino acids ISQII are required for D10’s colocalization with mitochondria.

10.1128/mbio.00300-22.4 FIG S4 D10 mutants with deletion of amino acids 9 to 13 or mutation expressed from recombinant VACV do not colocalize with mitochondria during infection, A549DKO cells were infected with indicated recombinant VACVs (MOI = 3) encoding D10 mutants with a C-terminal 3×Flag tag. Confocal microscopy was used to visualize D10 (α-Flag antibody, green), mitochondria (MitoTracker, red), and DNA (DAPI, blue) at 16 hpi. Download FIG S4, TIF file, 1.7 MB. Copyright © 2022 Cao et al. 2022 Cao et al. https://creativecommons.org/licenses/by/4.0/ This content is distributed under the terms of the Creative Commons Attribution 4.0 International license.

10.1128/mbio.00300-22.5 FIG S5 D10 mutants with deletion of amino acids 9 to 13 or mutation expressed from recombinant VACV do not colocalize with mitochondria during infection. HeLa cells were infected with indicated recombinant VACVs (MOI = 3) encoding D10 mutants with a C-terminal 3×Flag tag. Confocal microscopy was used to visualize D10 (α-Flag antibody, green), mitochondria (MitoTracker, red), and DNA (DAPI, blue) at 16 hpi. Download FIG S5, TIF file, 2.7 MB. Copyright © 2022 Cao et al. 2022 Cao et al. https://creativecommons.org/licenses/by/4.0/ This content is distributed under the terms of the Creative Commons Attribution 4.0 International license.

### Loss of D10 mitochondrial colocalization reduces VACV replication.

**FIG 3:**
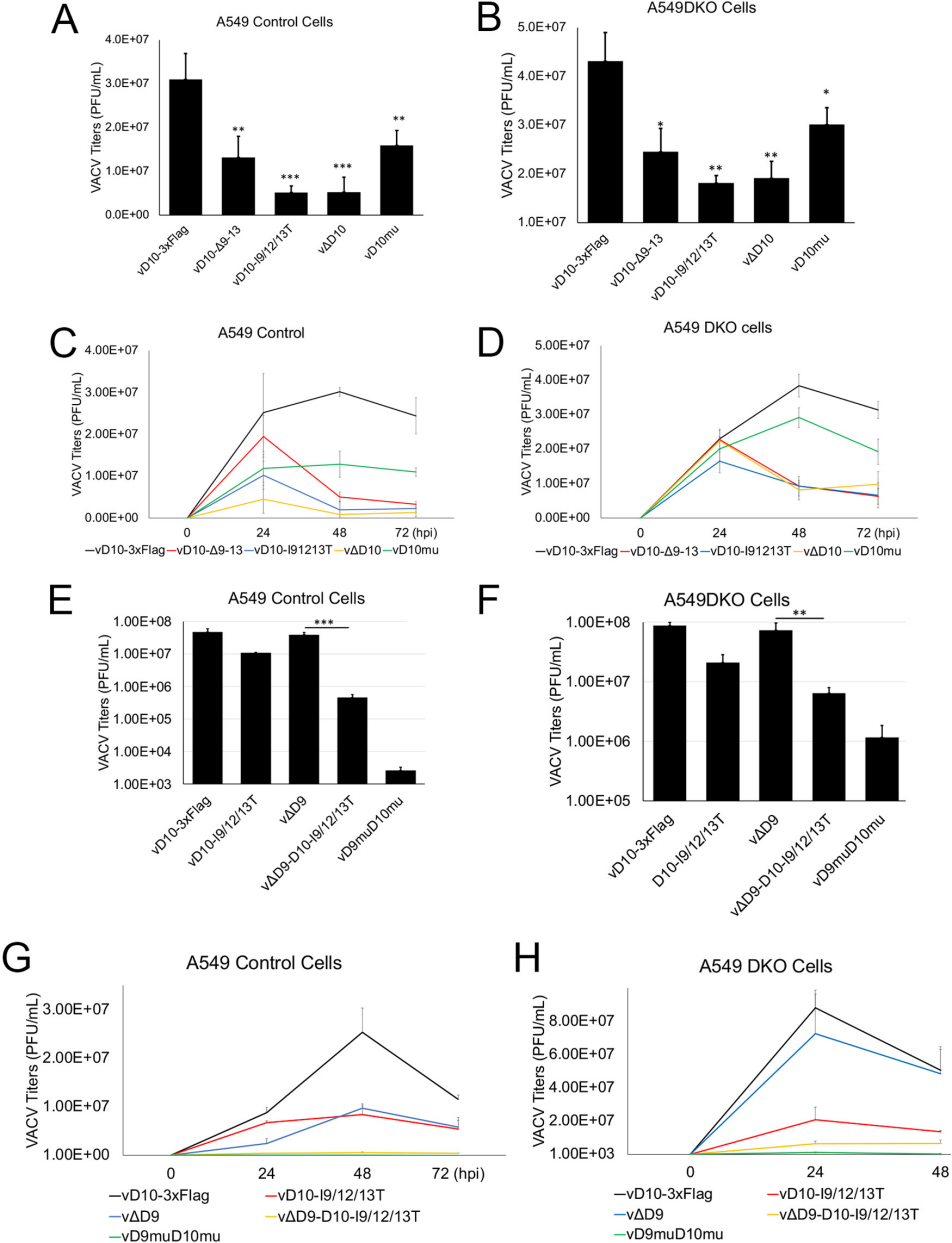
Loss of D10 mitochondrial colocalization reduces VACV replication. (A to D) Loss of D10 mitochondrial colocalization moderately impairs VACV replication in the presence of D9. A549 control (A and C) or A549DKO cells (B and D) were infected with indicated viruses at an MOI of 3 (A and B) or 0.001 (C and D). (E to H) Loss of D10 mitochondrial colocalization more substantially impairs VACV replication in the absence of D9 expression. A549 control (E and G) or A549DKO cells (F and H) were infected with indicated viruses at an MOI of 3 (E and F) or 0.001 (G and H). D9 was knocked out in vΔD9 and vΔD9-D10-I9/12/13T. Viral titers were determined using a plaque assay. Error bars represent the standard deviations of at least three replicates. ns, *P* > 0.05; *, 0.01 < *P* ≤ 0.05; **, 0.001 < *P* ≤ 0.01; ***, 0.0001 < *P* ≤ 0.001. Significance was compared to vD10-3xFlag (panels A to D) or vΔD9 (panels E to H).

Next, we examined the impact of D10 mitochondrial colocalization on VACV replication by comparing the replication of vD10-Δ9-13 and vD10-I9/12/13T to vD10-3xFlag, a control VACV encoding wild-type (WT) D10 with a 3×Flag tag at its C terminus. Two viruses with D10 knocked out (vΔD10) and the D10 Nudix motif inactivated (vD10mu) (31), respectively, were included in the experiments. We used both A549 control and A549DKO cells, as Liu et al. had shown that A549DKO cells could better support decapping enzyme-inactivated VACV replication (34). The A549 control cells were generated in parallel with A549DKO cells but with no PKR and RNase L knocked out (34). All the recombinant viruses with mutated or deleted D10 replicated at a lower rate, with the infection multiplicities of infection (MOIs) of 3 and 0.001, respectively. The vD10-I9/12/13T more closely mimicked vΔD10 with more severe effects (∼3-fold higher decrease in both A549 control and A549DKO cells, 6- versus 2.5-fold at MOI of 3 and up to 15- versus 5-fold at MOI of 0.001 at 48 h postinfection [hpi]) on viral yields and replication kinetics than that of vD10mu and vD10-Δ9-13 (Fig. 3A to D). In addition, the reductions of VACV replication for all the tested mutant viruses were more prominent in A549 control cells than in A549DKO cells (Fig. 3A to D). Interestingly, in BHK-21 cells, the decrease of D10 mutant viruses was similar to that in A549DKO cells, with more moderate effects (Fig. S6).

10.1128/mbio.00300-22.6 FIG S6 BHK-21 cells were infected with indicated viruses at an MOI of 3 (A) or 0.001 (B). Viral titers were determined using a plaque assay at indicated times postinfection. All the viruses used encode D9. Error bars represent the standard deviation of at least three replicates. ns, *P* > 0.05; *, *P* ≤ 0.05. Significance was compared to vD10-3xFlag. Download FIG S6, TIF file, 0.2 MB. Copyright © 2022 Cao et al. 2022 Cao et al. https://creativecommons.org/licenses/by/4.0/ This content is distributed under the terms of the Creative Commons Attribution 4.0 International license.

VACV encodes two decapping enzymes, D9 and D10, that have overlapping yet distinct functions (24, 25, 35). We rationalized that the loss of D10 mitochondrial colocalization has a more prominent effect on VACV replication in the absence of D9 expression. We generated a recombinant VACV vΔD9-D10-I9/12/13T, in which the D9 was knocked out, and compared its replication with vΔD9 (D9 knocked out with wild-type D10). We included vD10-3xFlag and vD9muD10mu (in which the decapping activities of both D9 and D10 are deactivated) (32) in this experiment. Consistent with a previous report (31), the replication of vΔD9 was not or only slightly affected in A549 control and A549DKO cells at an MOI of 3 or 0.001, while vD9muD10mu barely replicated in A549 control cells but could replicate at some levels in A549DKO cells (Fig. 3E to H). Notably, compared to vΔD9, vΔD9-D10-I9/12/13T showed an 83-fold and 11-fold reduction of viral yield at an MOI of 3 in A549 control and A549DKO cells, respectively (Fig. 3E and F). At an MOI of 0.001, vΔD9-D10-I9/12/13T replication also showed an 18-fold and 11-fold reduction of viral yields at its replication peaks in A549 control and A549DKO cells, respectively (Fig. 3G and H).

Overall, we conclude that D10 mitochondrial colocalization is required for efficient VACV replication in both PKR-and RNase L-dependent and independent manners.

### Loss of D10 mitochondrial colocalization reduces VACV plaque sizes.

**FIG 4:**
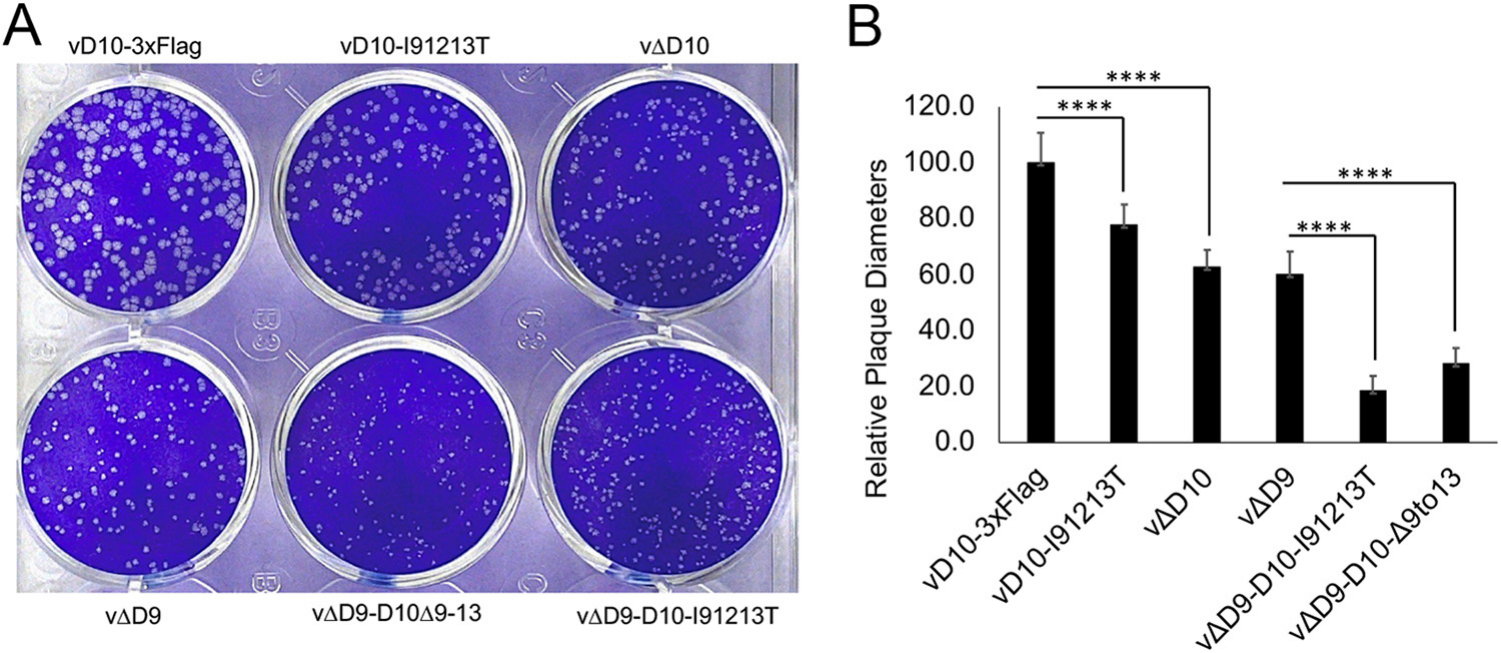
Loss of D10 mitochondrial colocalization significantly decreases VACV plaque sizes. (A) Loss of D10 mitochondrial colocalization reduced VACV plaque sizes. BS-C-1 cells were infected with indicated viruses. Plaques were visualized by a plaque assay. (B) Diameters from 25 plaques were measured using ImageJ and plotted. The diameters of vD10-3xFlag plaques were normalized to 100. ****, *P* ≤ 0.0001.

We next determined the impact of D10 mitochondria colocalization on viral plaque size in BS-C-1 cells. The results indicated that vΔD9-D10-I9/12/13T and vΔD9-D10Δ9-13 have significantly smaller plaques than vD10-3xFlag, as well as vΔD9 (Fig. 4A and B). The plaque sizes of vD10-I9/12/13T and vΔD10 were also smaller than vD10-3xFlag (Fig. 4A and B), suggesting an important role of D10 mitochondrial colocalization in VACV replication and spread to other cells.

### Loss of D10 mitochondrial colocalization reduces VACV protein levels in infected cells.

**FIG 5:**
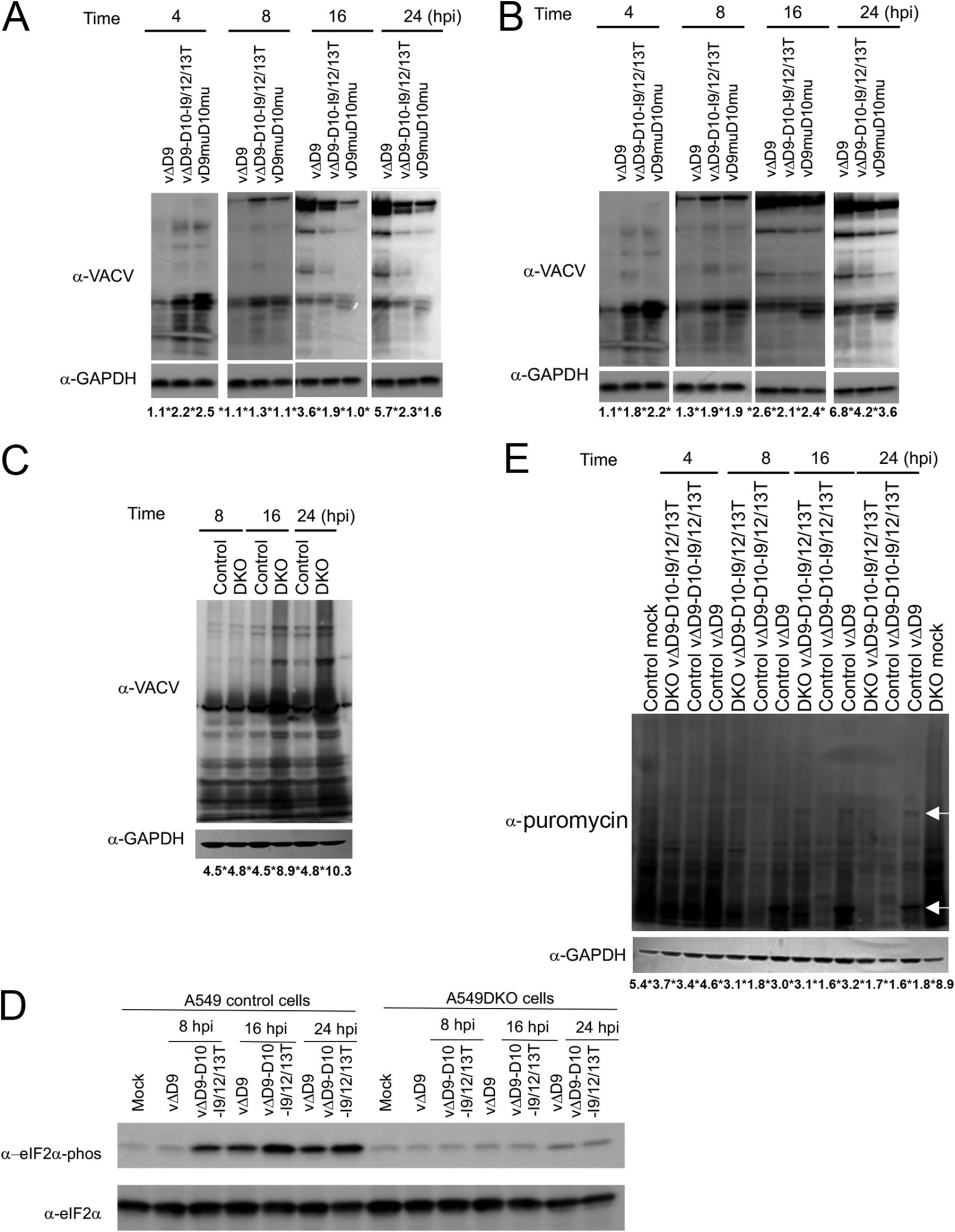
Loss of D10 mitochondrial colocalization impairs VACV protein synthesis. (A and B) A549 control (A) or A549DKO (B) cells were infected with indicated viruses at an MOI of 3. Viral proteins were detected using α-VACV serum at indicated times postinfection. GAPDH was used as a loading control. (C) A comparison of VACV protein levels in A549 control and A549DKO cells at the same time postinfection is shown. (D) Western blotting of eIF2α phosphorylation in A549 control and A549DKO cells infected with indicated viruses at indicated times postinfection. (E) A549 control or A549DKO cells were infected with indicated viruses at an MOI of 5 or mock infected. Cells were treated with 10 μg/mL puromycin for 20 min before collection at indicated time points, followed by Western blotting using α-puromycin and α-GAPDH antibodies. Arrows indicate two highly expressed VACV proteins. Figures shown in each panel are the representatives of three biological replicates. The numbers below each lane indicate GAPDH-normalized intensities. The asterisk is used to separate numbers.

Our results (Fig. 3) demonstrated that the effect of D10 mitochondrial colocalization on reducing VACV replication could be more readily observed in the absence of D9. We next compared viral protein levels of vΔD9, vΔD9-D10-I9/12/13T, and vD9muD10mu during infection. We observed lower levels of viral proteins in vΔD9-D10-I9/12/13T infection than that in vΔD9 infection, but more viral proteins than that in vD9muD10mu infection in A549 control cells at late times of infection (16 and 24 hpi) (Fig. 5A). Interestingly, there was a much smaller difference in protein levels in vΔD9-D10-I9/12/13T- and vΔD9-infected A549DKO cells (Fig. 5A and B). In fact, when we compared the viral protein levels of vΔD9-D10-I9/12/13T-infected A549 control and A549DKO cells in parallel over the course of infection, higher viral protein levels in A549DKO cells were observed at 16 and 24 hpi, but not at 8 hpi (Fig. 5C). The results indicate that, similar to vD9muD10mu (although with less extent), the A549DKO cells were more permissive than the A549 control cells to vΔD9-D10-I9/12/13T infection.

It has been shown that inactivation of D9 and D10 decapping activities stimulate PKR activation, followed by eIF2α phosphorylation (32), leading to translational repression. One of the underlying mechanisms of the higher permissiveness to vΔD9-D10-I9/12/13T infection in A549DKO cells could be attributed to less restriction on nascent protein synthesis than that in A549 control cells, similar to vD9muD10mu infection (34). We compared eIF2α phosphorylation in vΔD9- and vΔD9-D10-I9/12/13T-infected cells and observed higher eIF2α phosphorylation in vΔD9-D10-I9/12/13T-infected A549 control cells, but not A549DKO cells (Fig. 5D). It is known that VACV infection causes a global protein synthesis shutoff of host cells (host shutoff) (41). Puromycin labeling of nascent protein synthesis indicated that vΔD9-D10-I9/12/13T infection of A549 control cells still caused a profound host shutoff, which was even more rapid than that in vΔD9-D10-I9/12/13T infection of A549DKO cells and vΔD9 infection of A549 control cells (Fig. 5E). The result was consistent with the high eIF2α phosphorylation in vΔD9-D10-I9/12/13T-infected A549 control cells (Fig. 5D). Viral proteins are selectively synthesized during the host shutoff. Our results also indicated that viral proteins were synthesized at a lower rate in vΔD9-D10-I9/12/13T-infected A549 control cells than in vΔD9-D10-I9/12/13T infection of A549DKO cells and vΔD9 infection of A549 control cells (Fig. 5E). Together, these results demonstrate that loss of mitochondrial colocalization decreases VACV protein synthesis.

### Loss of mitochondrial localization reduces D10’s gene expression shutoff ability.

**FIG 6:**
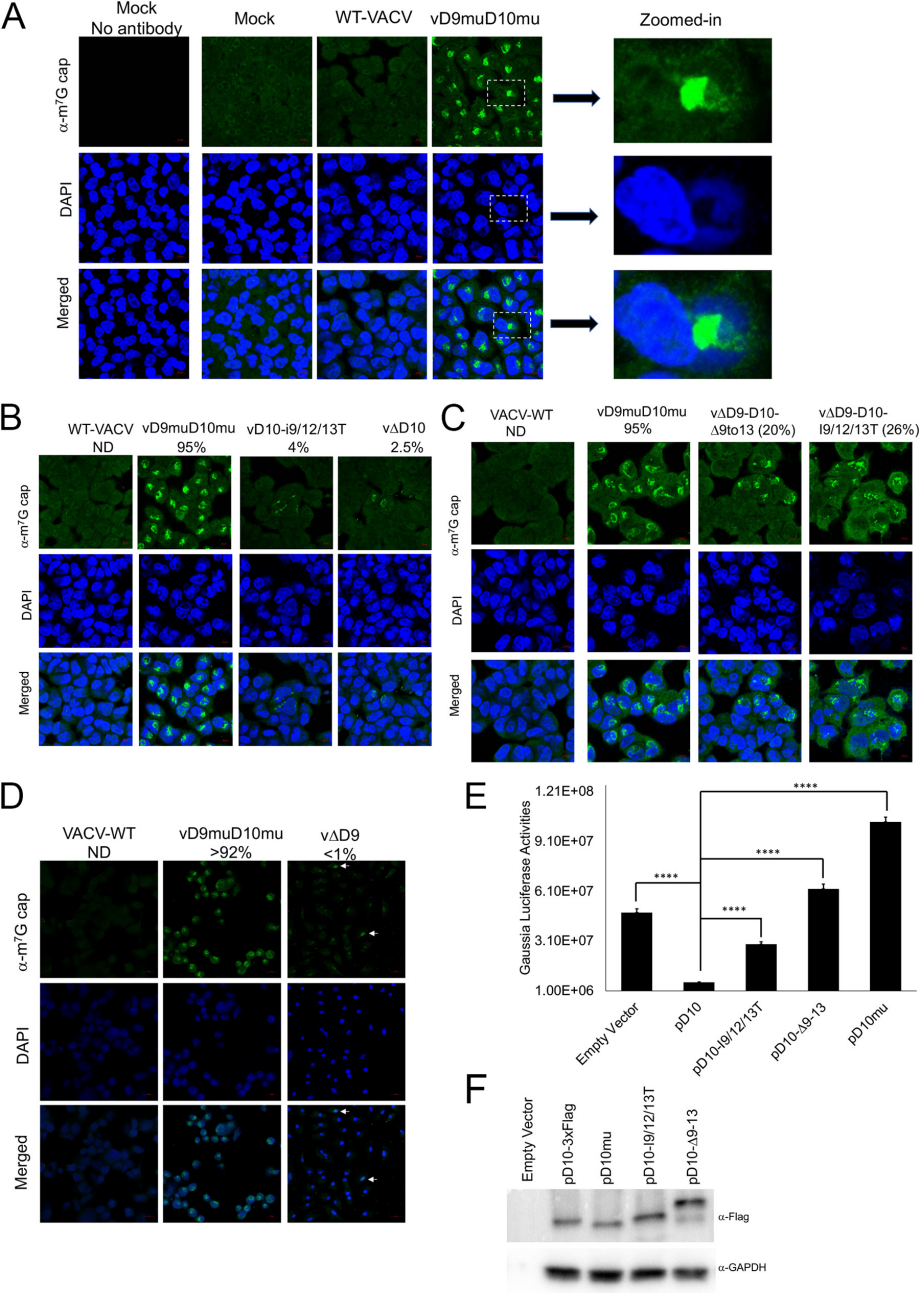
Loss of mitochondrial colocalization reduces D10’s gene expression shutoff capability. (A) Inactivation of D9 and D10’s decapping activities leads to highly accumulated m^7^G caps between viral factories and nuclei in VACV-infected cells. A549DKO cells were infected with WT or vD9muD10mu (MOI = 3) or mock infected. Confocal microscopy was used to visualize m^7^G cap (α-cap antibody, green) and DNA (DAPI, blue) at 16 hpi. Three zoomed-in areas were shown on the right. (B and C) Loss of mitochondrial colocalization leads to regions with highly accumulated caps in the cytoplasm of VACV-infected cells. A549DKO cells were infected with indicated viruses at an MOI of 3. Confocal microscopy was used to visualize m^7^G cap (α-cap antibody, green) and DNA (DAPI, blue) at 16 hpi. The numbers indicate the percentages of cells containing regions with accumulated caps from at least six views. (D) vΔD9 infection does not lead to highly accumulated cap regions in the cytoplasm of VACV-infected cells. A549DKO cells were infected with indicated viruses at an MOI of 3. Confocal microscopy was used to visualize m^7^G cap (α-cap antibody, green) and DNA (DAPI, blue) at 16 hpi. The numbers indicate the percentages of cells containing regions with accumulated caps in the cytoplasm from multiple randomly pictured views. Arrows indicate two cells with an increase of cap staining in the nuclei of vΔD9-infected cells. (E) Loss of D10 mitochondrial colocalization reduces its ability to shut off gene expression. Plasmid encoding a *Gaussia* luciferase reporter gene under a cellular EF-1α promoter was cotransfected with the indicated plasmids encoding codon-optimized D10 or D10 mutants. *Gaussia* luciferase activities were measured 24 h posttransfection. (F) Western blotting of D10 and D10 mutant protein levels (a representative image of three biological repeats). Error bars represent the standard deviation of at least three replicates. ****, 0.001 < *P* ≤ 0.0001.

Our results in Fig. 5D indicated that vΔD9-D10-I9/12/13T infection of A549 control cells strongly stimulated eIF2α phosphorylation, suggesting that the loss of mitochondrial colocalization may impair its decapping activity in cells and lead to reduced RNA degradation. We employed an immunofluorescence assay to visualize endogenous 5′-capped mRNAs in cells using α-cap antibodies. We used A549DKO cells, as they support the replication of VACV with inactivated decapping enzymes, and the mRNAs are not degraded through the RNase L pathway (34). In mock- and WT VACV-infected A549DKO cells, the caps distributed in the cells mostly evenly (Fig. 6A). Interestingly, we observed similar intensities in mock and wild-type (WT) VACV-infected cells, suggesting that VACV produced capped mRNAs in the infected cells, while cellular mRNAs were decapped. Strikingly, highly accumulated 5′-cap staining was observed in subcellular regions of almost all cells infected with vD9muD10mu. The highly accumulated regions with cap staining were located primarily at the perinucleus regions between the nuclei and viral factories (VACV DNA replication site in the cytoplasm with intensive DAPI [4′,6-diamidino-2-phenylindole] staining) (Fig. 6A). The accumulation of 5′ caps indicates the inability of vD9muD10mu to remove RNA 5′ cap during infection. We then investigated the impacts of mitochondrial colocalization loss on m^7^G-capped RNA in the presence or absence of D9 expression during VACV infection (Fig. 6B and C). In the presence of D9, loss of D10 mitochondrial colocalization or D10 deletion slightly increased the number of cells containing regions with accumulated 5′ caps, with 4% and 2.5% of cells containing regions with accumulated caps (Fig. 6B). Notably, in the absence of D9 expression, the loss of D10 mitochondrial colocalization substantially increased the regions with accumulated caps during infection, with 26% and 20% of cells for vΔD9-I9/12/13T and vΔD9-ΔD109-13, respectively (Fig. 6C). As a control, vΔD9 infection of A549DKO cells barely induced the highly accumulated cap regions in the cytoplasm (<1%) (Fig. 6D); however, it led to an increase of 5′-cap staining in the nuclei of some cells (Fig. 6D), suggesting that D9 plays a role to remove RNA 5′ cap in nuclei, an intriguing phenotype prompting further investigation of the different roles of D9 and D10 in subcellular organelles during infection.

These results (Fig. 5 and Fig. 6B) suggest that mitochondrial colocalization is required for efficient decapping in cells, which leads to RNA degradation and gene expression shutoff. We employed a virus-free approach to testing if loss of mitochondrial colocalization impairs D10's ability to induce gene expression shutoff in cells without interference from other viral factors. We cotransfected plasmids encoding codon-optimized D10 or its mutants with a *Gaussia* luciferase reporter plasmid under a cellular EF-1α promoter. As expected, WT D10 potently decreased *Gaussia* luciferase activity by 7.8-fold (Fig. 6E). However, cotransfection of a plasmid expressing D10-I9/12/13T could only reduce Gaussian luciferase expression by 1.7-fold (Fig. 6E). D10Δ9-13 and D10mu (with Nudix domain mutation) lost their ability to suppress *Gaussia* luciferase expression (Fig. 6E). The protein expression levels of D10 and its mutants from plasmids were comparable. Interestingly, the D10Δ9-13 also showed a slower migration band when expressed from a plasmid (Fig. 6F), but not from the recombinant virus (Fig. 2D). The slower D10Δ9-13 migration band was reproducibly observed when expressed from the plasmid. Although we do not know the nature of this band, it likely represents a posttranslational modification of this mutant protein in uninfected cells. Taken together, our results indicate that loss of mitochondrial colocalization reduces D10's ability to remove mRNA 5′ caps and shut off gene expression.

### Loss of mitochondrial colocalization impairs D10’s mRNA translation enhancement ability.

**FIG 7:**
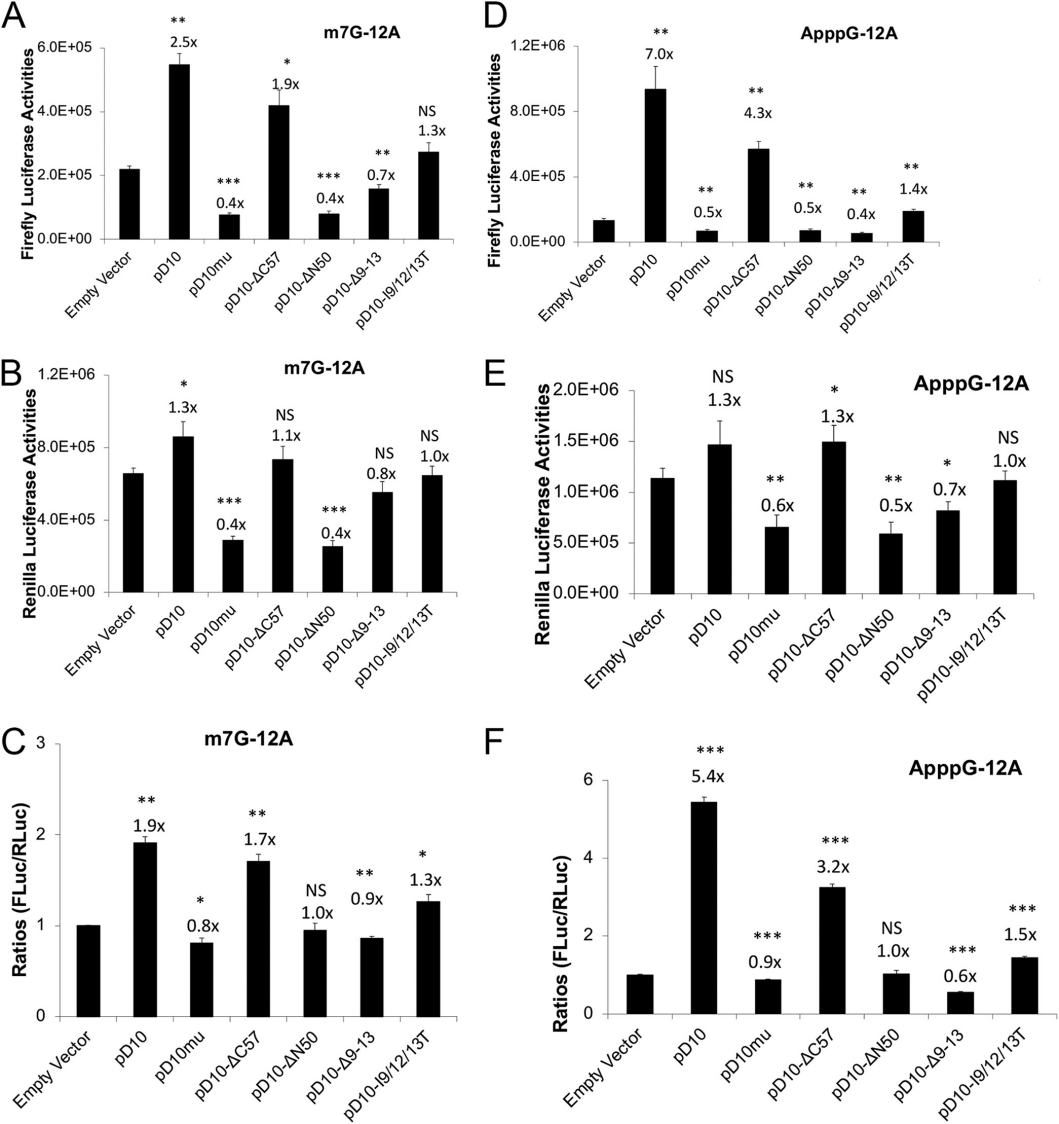
Loss of mitochondrial colocalization reduces D10’s mRNA translation enhancement ability for both cap-dependent and cap-independent translation. (A to C) 293T cells were transfected with indicated plasmids. Forty-two hours posttransfection, *in vitro*-synthesized, m^7^G-capped 12A-Fluc and Kozak-Rluc were cotransfected into the 293T cells. Luciferase activities were measured 6 h post-RNA transfection. Fluc (A), Rluc (B), and Fluc/Rluc ratios with the empty vector normalized to 1 (C) are presented. (D to F) 293T cells were transfected with indicated plasmids. Forty-two hours posttransfection, *in vitro*-synthesized, ApppG-capped 12A-Fluc and Kozak-Rluc (m^7^G-capped) were cotransfected into the 293T cells. Luciferase activities were measured 6 h post-RNA transfection. Fluc (D), Rluc (E), and Fluc/Rluc ratios with the empty vector normalized to 1 (F) are presented. Error bars represent the standard deviation of three replicates. Significance determined by Student's *t* test where *P* > 0.05 (ns), *, 0.01 < *P* ≤ 0.05; **, 0.001<*P* ≤ 0.01; ****, 0.0001 < *P* ≤ 0.001. The numbers above significance represent fold changes. Significance and fold changes were compared to the empty vector.

D10 expression promotes mRNA translation, especially for mRNAs with a 5′ poly(A) leader, a feature of all poxvirus mRNAs expressed after viral DNA replication (35). The enhancement is more notable for RNA without a 5′ m^7^G cap and could be revealed in the absence of VACV infection (35). We employed an RNA-based luciferase reporter described previously (42, 43). We used 293T cells in these experiments, as we found this cell line had the highest transfection efficiency in the cells we tested. *In vitro*-transcribed firefly luciferase (FLuc) RNA with a 5′ poly(A) leader and m^7^G cap and renilla luciferase (RLuc) RNA with a 5′ untranslated region (UTR) containing Kozak sequence and m^7^G capped were cotransfected in cells with expression of wild-type D10 or its mutants. Notably, all those containing mitochondrial colocalization sequences promoted 5′-poly(A) leader-mediated translation, while those without mitochondrial colocalization sequences significantly reduced the translation enhancement (Fig. 7A to C). ApppG-capped RNA translation only occurs in a cap-independent manner (44, 45). The same trends were observed when ApppG-capped, 5′ poly(A) leader Fluc mRNA was used, although the translation enhancement was much higher than m^7^G-capped RNA (Fig. 7D to F). Interestingly, D10mu even decreased translation of both m^7^G- and ApppG-capped, 12A-headed reporter mRNA (Fig. 7). This may be due to the complete loss of D10’s decapping activity, leading to less translation machinery available for the reporter mRNA’s translation. Together, our results show that the mitochondrial colocalization is required for D10 to stimulate 5′ poly(A) leader mRNA translation, including cap-independent translation enhancement.

## DISCUSSION

In this study, we identified and characterized the first mitochondria-colocalized decapping enzyme, D10, encoded by a poxvirus. We pinpointed three hydrophobic isoleucine residues at the N terminus of D10 that are essential for D10’s mitochondrial colocalization. The mitochondrial association is required for D10’s unusual function to promote 5′ poly(A) leader-mediated mRNA translation, including cap-independent translation enhancement. The mitochondrial colocalization is also necessary for D10's optimal function to efficiently remove 5′ caps of RNAs in cells and shut off gene expression. Consequently, mitochondrial colocalization is required for efficient VACV replication. Interestingly, one group of mRNAs that are preferentially targeted by D10 for degradation are those involved in oxidative phosphorylation, which was reported by Ly et al. during the revision of this manuscript (46). As mRNA-encoded oxidative phosphorylation complex proteins are often translated by ribosomes associated with mitochondria (47, 48), it further supports the close association of D10 with mitochondria. At this point, we do not know the biochemical and biophysical nature of D10 and mitochondria association. In fact, we did not observe copurification of D10 with mitochondria in attempting to examine if D10 was able to be copurified with mitochondria after lysis of the cells (not shown). The results suggest that the association between D10 and mitochondria is disrupted after the cells were lysed. One possibility is that D10 colocalization with mitochondria requires cells to be alive and intact. Although the nature and mechanism of D10 and mitochondria colocalization in live cells still need further investigation, D10 likely resides on mitochondria through interacting with a component on mitochondrial outer membrane or surface, such that its catalytically active Nudix decapping motif is exposed to the cytoplasm to remove 5′ caps of mRNAs. Although the mitochondria association is required for the optimal decapping activity of D10 in live cells, the N-terminal mutation is not part of the Nudix decapping motif’s intrinsic enzymatic activity, as the Nudix motif locates in the center region of D10, which is over 120 amino acids away from the mutation site. Further study will investigate the molecular mechanism by which D10 regulates its decapping activity through associating with mitochondria.

**FIG 8:**
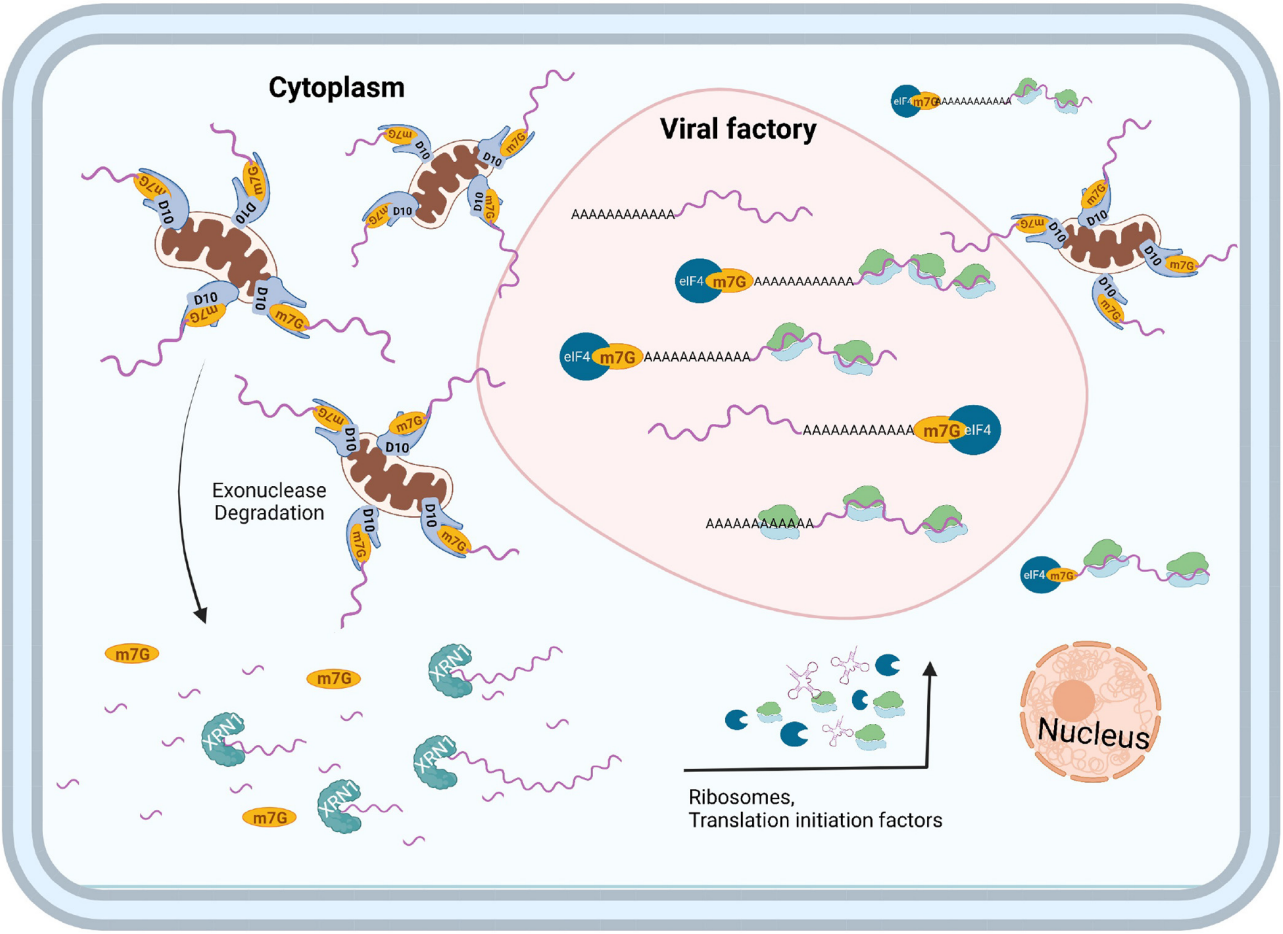
Model of how D10 mitochondria localization impacts its functions. By localizing to mitochondria, D10 (i) preferentially decaps cellular cytoplasmic mRNAs, (ii) concentrates locally for powerful decapping activity, (iii) rapidly mobilizes in the cytoplasm to access RNA substrates, (iv) assembles decapping and mRNA degradation complex, and (v) frees up and restricts its competition with translation machinery.

There are several possible yet nonexclusive mechanisms by which mitochondrial colocalization is required for the optimal effect of D10 to shut off gene expression (Fig. 8). First, D10 may need to assemble decapping and/or mRNA degradation complex comprising other cellular or viral proteins around mitochondria to efficiently perform their function. Second, parking on mitochondria efficiently concentrates D10 locally, which could amplify the decapping efficiency of D10. In fact, mounting evidence shows that many proteins can concentrate for maximal effects, such as phase separation (49). For decapping enzymes, one model of Dcp2 function is through concentrating decapping and RNA degradation complex in P-bodies for efficient mRNA decay (14, 15, 50). Third, mitochondria serve as the highly dynamic vehicles to transport the associated D10 throughout the cytoplasm to access mRNAs more readily, given that mitochondria are highly mobile organelles (51, 52). Fourth, mitochondrial colocalization may be required for proper conformation of D10 to remove the RNA 5′ cap efficiently. Further investigations of these possibilities are ongoing.

How D10’s mitochondrial association facilitates mRNA translation, especially cap-independent translation enhancement, is thought-provoking but needs extensive investigation of D10’s molecular functions in the presence and absence of VACV infection. As inactivation of its decapping activity also renders it to lose its ability to promote translation (35), the mitochondrial colocalization requirement for translation promotion could be due to its reduced ability to induce mRNA degradation to release translation machinery in cells. In the meantime, the mitochondrial colocalization may restrict its ability to interfere with ribosome recruitment by mRNAs, either in a cap-dependent or a cap-independent manner. Because both decapping activity and mitochondria association are needed for translation promotion, these two functions likely promote mRNA translation in a synergistic manner.

In addition to being required for optimal gene expression shutoff and translation promotion, D10's mitochondrial colocalization may provide additional mechanisms to promote VACV replication in VACV-infected cells. Notably, it provides a spatial mechanism for D10 to more readily decap cellular mRNAs (Fig. 8). Viral factories are the sites for viral DNA transcription where the viral transcripts are actively translated to produce proteins (53). As mitochondria are not found in the viral factories (Fig. 1 and 2), D10 does not decap the viral mRNAs in the factories, especially those postreplicative mRNAs transcribed after viral DNA replication. In contrast, cellular mRNAs and early viral mRNAs transcribed before viral factories formation are more likely accessed by D10 for decapping and subsequent degradation through the highly mobile mitochondria. We and others previously observed pervasive transcription initiation of the VACV genome, especially during the late time of replication (54–57). These transcripts are from both sense and antisense strands of the viral genome. Many of these transcripts are small but still get capped. More importantly, many of them form dsRNA. As these RNAs are less likely to be loaded by ribosomes and get translated, they more likely escape from viral factories to be accessed by D10 on mitochondria. Interestingly, human Dcp2 is predominantly in the cytoplasm, with many concentrated in P-bodies, particularly under stress (14, 15, 19, 37). NUDT16 mainly localizes in nuclei, especially in nucleoli (21), suggesting its main function is to regulate nucleolar RNAs. A decapping enzyme from Africa swine fever virus mainly localizes to the endoplasmic reticulum and colocalizes with RNA caps (58). NUDT12 localizes to a few discrete cytoplasmic granules distinct from P-bodies for cytoplasmic surveillance of NAD-capped RNAs (22). These studies suggest diverse strategies that decapping enzymes use for their functions, demanding further investigation of these fascinating proteins.

D10 mitochondria association may affect VACV replication in addition to regulating protein synthesis in VACV infection. While in A549 control cells, VACV protein synthesis rate and level decreased (Fig. 5); a linear link between viral protein synthesis and viral titer should not be assumed, as VACV contains dozens of virion proteins, and these proteins may be differentially affected by the loss of D10 mitochondria association. In fact, in A549DKO cells, although the protein synthesis was not substantially affected (Fig. 5), VACV yields were significantly affected (Fig. 3), suggesting the involvement of post-protein synthesis events. One possibility is D10’s mitochondrial colocalization is required for viral spread, supported by the significantly smaller plaques formed by loss of mitochondria association with VACV D10 mutant viruses (Fig. 4).

In summary, our study identified a spatial mechanism for a poxvirus-encoded decapping enzyme to regulate mRNA metabolism and translation, resulting in an important role in viral replication. This study also provides a new strategy a decapping enzyme uses to regulate its function by riding mitochondria. As decapping enzymes are a group of diverse proteins with important physiological functions, it is of great interest to further dissect the molecular mechanisms.

## MATERIALS AND METHODS

### Cells and viruses.

A549 control cells and A549DKO cells (kind gifts from Bernard Moss) (34), human foreskin fibroblasts (HFFs) (a kind gift from Nicholas Wallace), HeLa cells (ATCC CCL-2), 293T cells (ATCC CRL-3216), and BHK-21 cells (C-13) were cultured in Dulbecco’s minimal essential medium (DMEM; Quality Biological). BS-C-1 cells (ATCC CCL-26) were cultured in Eagle’s minimal essential medium (EMEM; Quality Biological). The cell culture media were supplemented with 10% fetal bovine serum (FBS; Peak Serum), 2 mM glutamine (Quality Biological), 100 U/mL of penicillin (Quality Biological), and 100 μg/mL streptomycin (Quality Biological). Cells were grown at 37°C with 5% CO_2_.

VACV Western Reserve (WR) strain (ATCC VR-1354) is used in this study. Other recombinant VACVs used in this study were derived from the VACV WR strain. vD9muD10mu, vD10mu, vΔD10, and vΔD9 were kindly provided by Bernard Moss and described elsewhere (31, 32). vD10-3xFlag-expressing VACV D10 with a 3×Flag tag at the C terminus was described previously (35). Recombinant VACVs carrying mutant D10, including vD10-Δ9-13, vD10-I9/12/13T, vΔD9-D10-Δ9to13, and vΔD9-D10-I9/12/13T, were generated through homologous recombination using DNA fragments carrying indicated mutations, respectively, followed by three to four rounds of plaque purification of the recombinant viruses.

VACV and its derived recombinant viruses were grown in HeLa or A549DKO cells and purified on a 36% sucrose cushion. The viruses (except for vD9muD10mu) were titrated using a plaque assay as described elsewhere (59). The vD9muD10mu was titrated in A549DKO cells as described elsewhere using anti-VACV antibody immune staining (32, 59).

### Virus infection and plaque assay.

Virus infection was carried out with DMEM or EMEM containing 2.5% FBS. Virus was sonicated and diluted according to the desired MOI. Medium containing viruses was added to the cultured cells and incubated at 37°C for 1 h and replaced with fresh medium. For plaque assay, virus-containing samples were 10-fold serial diluted and added on top of BS-C-1 cells in 12-well plates. After 1 h of incubation at 37°C, the medium was replaced with fresh medium containing 0.5% methylcellulose (Fisher Scientific). Plaques were visualized by staining the infected cells in 12-well plates with 20% ethanol containing 0.1% crystal violet for 5 min.

To compare plaque sizes, the diameters of 25 representative plaques of each virus were picked and measured with ImageJ software.

### Antibodies and chemicals.

Mouse α-Flag monoclonal antibody (used for Western blotting) was purchased from Sigma-Aldrich (catalog no. F3165). Rabbit α-Flag polyclonal antibody (used immunostaining for confocal microscopy) was purchased from Thermo Fisher Scientific (catalog no. PA1-984B). Mouse α-Tom20 antibody (catalog no. sc-17764) and mouse α-GAPDH (glyceraldehyde-3-phosphate dehydrogenase) antibody (catalog no. sc-365062 HRP) were purchased from Santa Cruz Biotechnology. Rabbit α-VACV and rabbit α-A7 antibodies were kindly provided by Bernard Moss (60). Mouse α-cap antibody (catalog no. 201-001) was purchased from Synaptic Systems. MitoTracker (catalog no. M7510) was purchased from Thermo Fisher Scientific. Puromycin (catalog no. 10191-150) was purchased from VWR, and α-puromycin antibody (catalog no. MABE343) was purchased from Sigma-Aldrich.

### Plasmids and transfection.

Plasmids encoding D10 mutants are illustrated in Fig. 2A and include pD10-ΔN8, pD10-ΔN13, pD10-ΔN34, pD10-ΔN50, pD10-ΔC57, pD10-Δ9-13, and pD10-I9/12/13T. These plasmids were generated using Q5 site-directed mutagenesis kit (New England Biolabs; catalog no. E0554) based on the previously described codon-optimized pD10-3×Flag according to the manufacturer’s protocol (35). According to the manufacturer's instructions, plasmid transfection was carried out using Lipofectamine 2000 (Thermo Fisher Scientific; catalog no. 11668019).

### Nascent protein synthesis analysis.

A puromycin labeling-based method to detect newly synthesized proteins was used as previously described (61). Briefly, 10 μg/mL of puromycin (Sigma-Aldrich) was added to the cells 20 min prior to sample collection. The cells were harvested for immunoblotting using anti-puromycin antibody.

### Western blot analysis.

Western blotting was performed as described previously (62). Briefly, the samples were resolved by sodium dodecyl sulfate-polyacrylamide gel electrophoresis (SDS-PAGE), followed by transferring to a polyvinylidene difluoride (PVDF) membrane. The PVDF membrane was blocked with 2% bovine serum albumin (BSA) or 5% milk plus 1% BSA at room temperature for 1 h and then incubated with primary antibodies in the same blocking buffer at room temperature for 1 h or at 4°C overnight. After washing with Tris-buffered saline with Tween 20 (TBST) three times, membranes were incubated with secondary antibodies at room temperature for 1 h and washed three times with TBST. Before imaging, the membrane was developed using SuperSignal West Femto maximum-sensitivity substrate (Thermo Fisher Scientific; catalog no. 34094). Antibodies were stripped from the membrane by Restore Western blot stripping buffer (Thermo Fisher Scientific; catalog no. 21059) for analysis using another antibody.

### Immunostaining and confocal microscope.

Mock, VACV-infected, or plasmid-transfected cells were fixed with 4% paraformaldehyde solution for 30 min at room temperature. The cell membrane was penetrated with 1× phosphate-buffered saline (PBS) containing 0.5% Triton X-100 for 10 min following being blocked with 1× PBS containing 2% BSA for 1 h. Primary antibodies were diluted in PBS (with 2% BSA) and incubated with cells for 1 h at room temperature. After three times of washing with 1× PBS, cells were incubated with secondary Alexa Fluor (488 nm for green and 594 nm for red)-conjugated IgG diluted in 1× PBS (with 2% BSA) at room temperature for 1 h. After three times of washing with 1× PBS, cells were stained with DAPI for 5 min and washed with 1× PBS two more times. Coverslips were mounted using 40% glycerol. Zeiss 880 or Zeiss 700 confocal microscopy was used to visualize the cells.

### *In vitro* RNA synthesis, transfection, and luciferase assay.

Synthesis of RNA *in vitro* was carried out as previously described using HiScribe T7 Quick high-yield RNA synthesis kit (New England Biolabs; catalog no. E2050) (35, 42, 43). The RNAs were cotranscriptionally capped with m^7^G anti-reverse cap analog or ApppG cap analog (New England Biolabs; catalog nos. 1411 and 1406). The RNAs were purified using a PureLink RNA minikit (Thermo Fisher Scientific; catalog no. 12183025) and transfected into cells using Lipofectamine 2000 (Thermo Fisher Scientific; catalog no. L11668019) according to the manufacturer's instructions. Six hours posttransfection, cell lysates were collected, and luciferase activities were measured using a dual-luciferase reporter assay system (Promega; catalog no. E1960) and GloMax Navigator microplate luminometer with dual injectors (Promega) as per manufacturer protocol.

### *Gaussia* luciferase assay.

The *Gaussia* luciferase activities were measured using a luminometer using the Pierce *Gaussia* luciferase flash assay kit (Thermo Scientific; catalog no. 16158).

### Statistical analysis.

The Student's *t* test was performed to evaluate statistical differences from at least three replicates. We used the following convention for symbols to indicate statistical significance: ns, *P > *0.05; *, 0.01 <* P ≤ *0.05; **, 0.001 <* P ≤ *0.01; ***, 0.0001 <* P ≤ *0.001; and ****, *P ≤ *0.0001.
