# Supplementary figures and images for: A Poxvirus Decapping Enzyme Colocalizes with Mitochondria To Regulate RNA Metabolism and Translation and Promote Viral Replication
Source: mBio. 2022 Apr 18;13(3):e00300-22. doi: 10.1128/mbio.00300-22 (PMC9239241; doi:10.1128/mbio.00300-22)

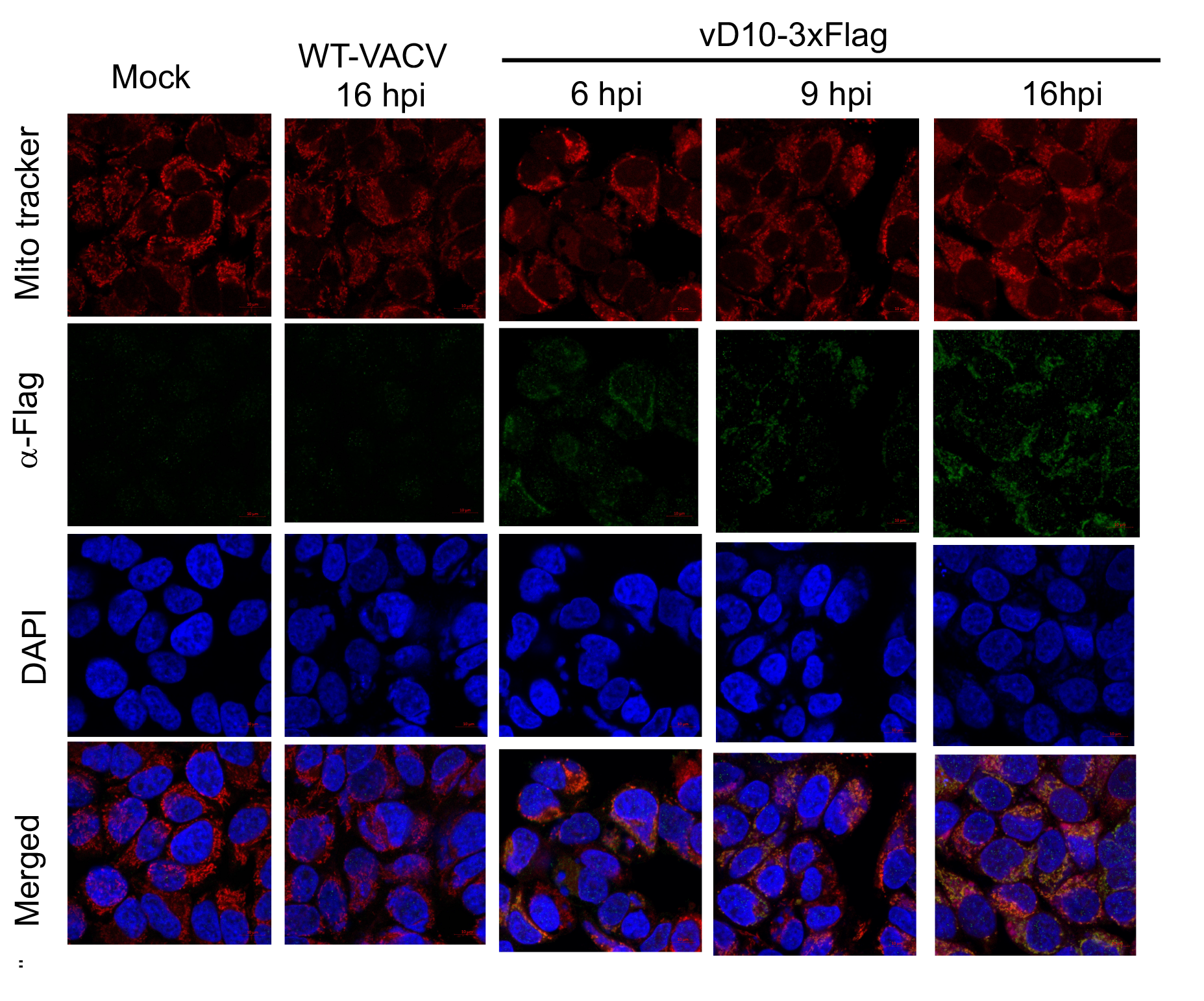

Supplement: FIG S1 [file mbio.00300-22-s0001.tif]

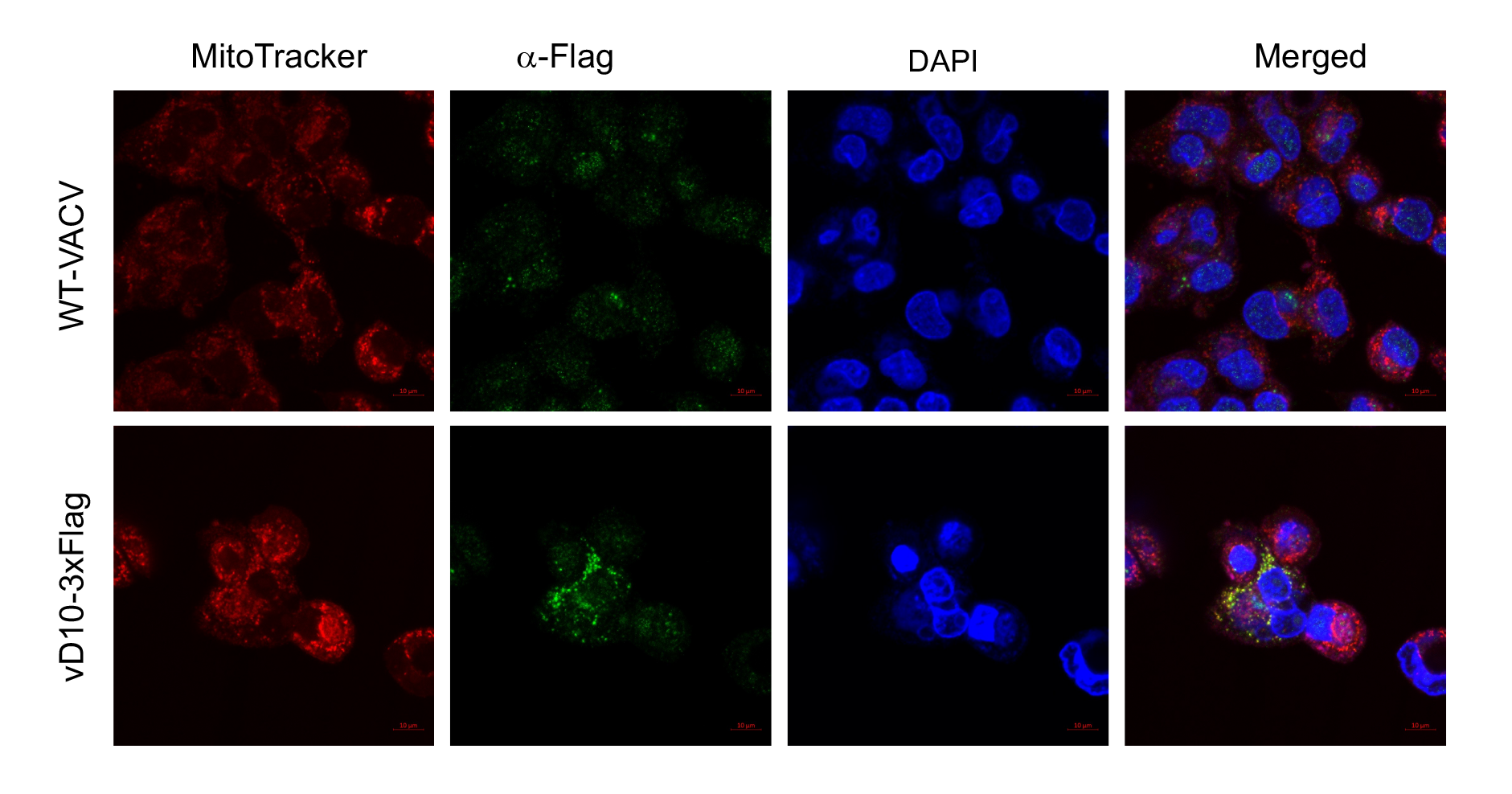

Supplement: FIG S2 [file mbio.00300-22-s0002.tif]

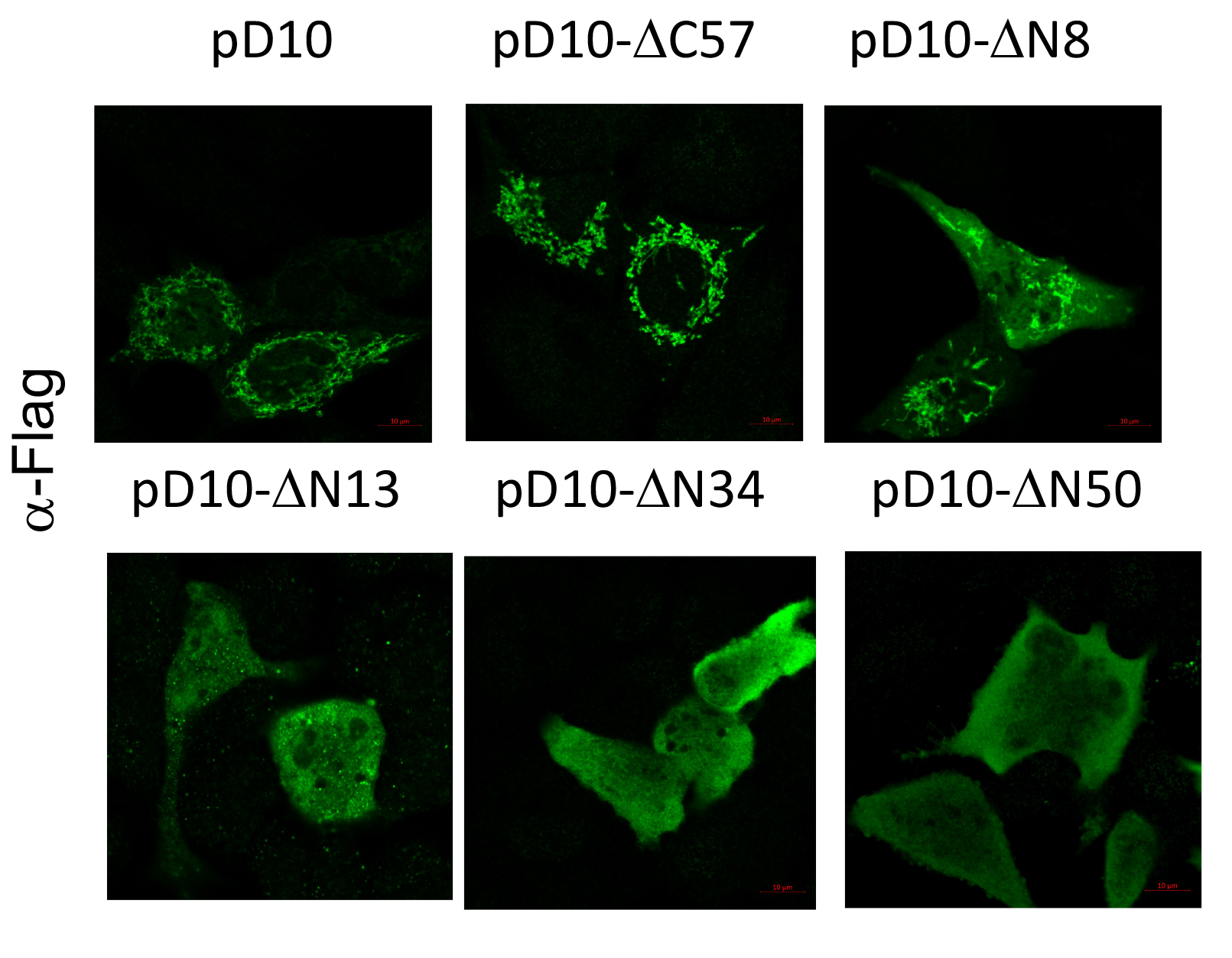

Supplement: FIG S3 [file mbio.00300-22-s0003.tif]

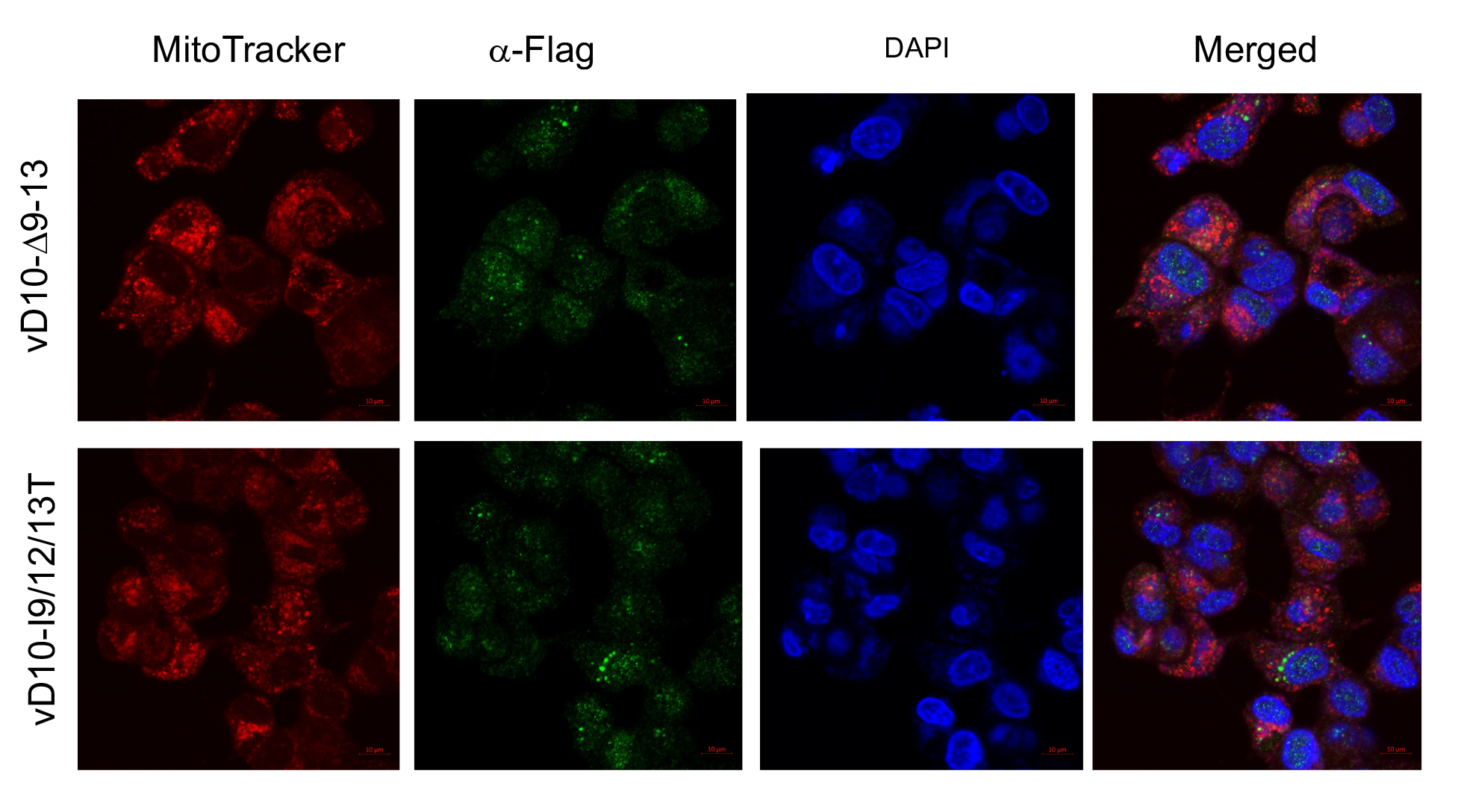

Supplement: FIG S4 [file mbio.00300-22-s0004.tif]

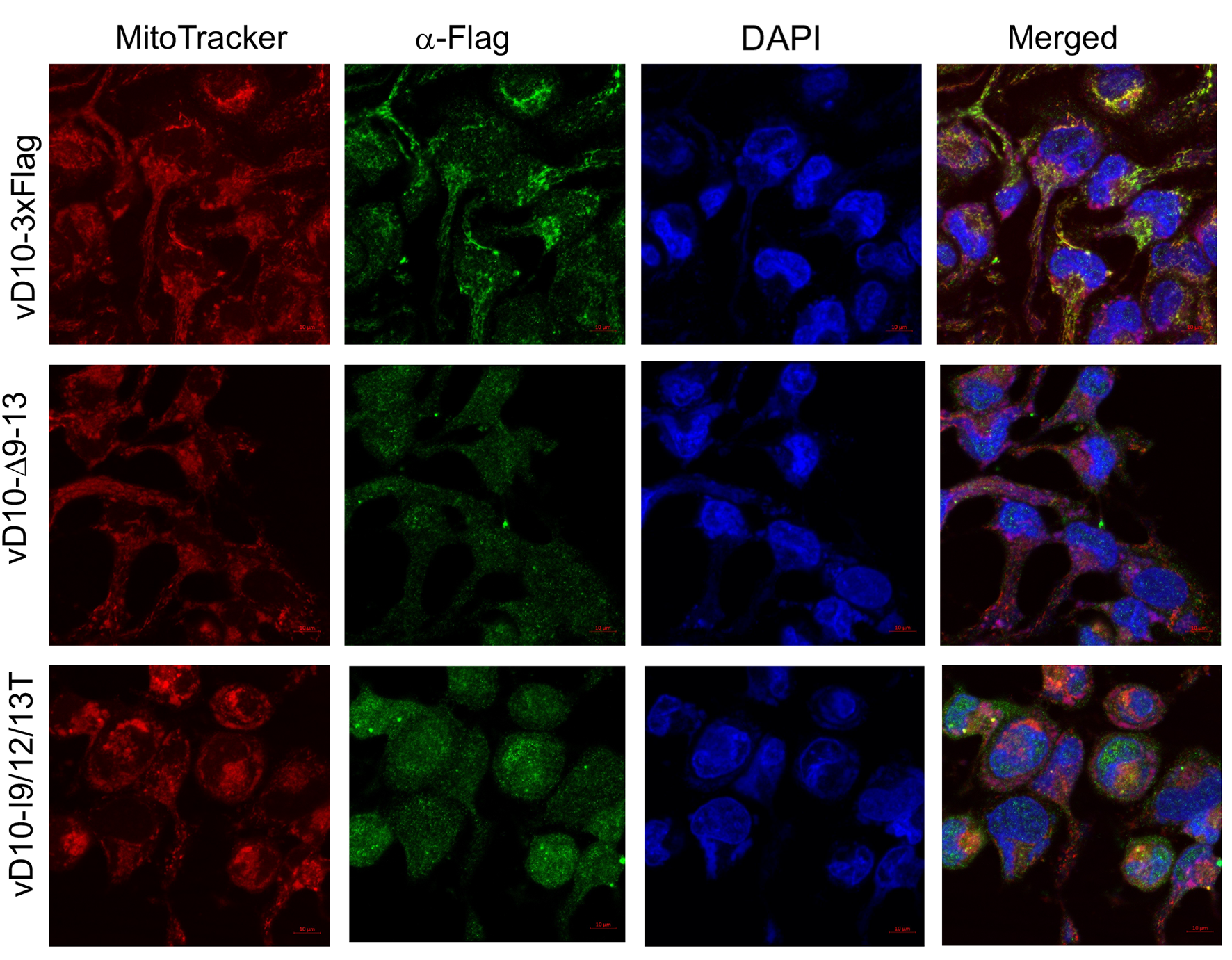

Supplement: FIG S5 [file mbio.00300-22-s0005.tif]

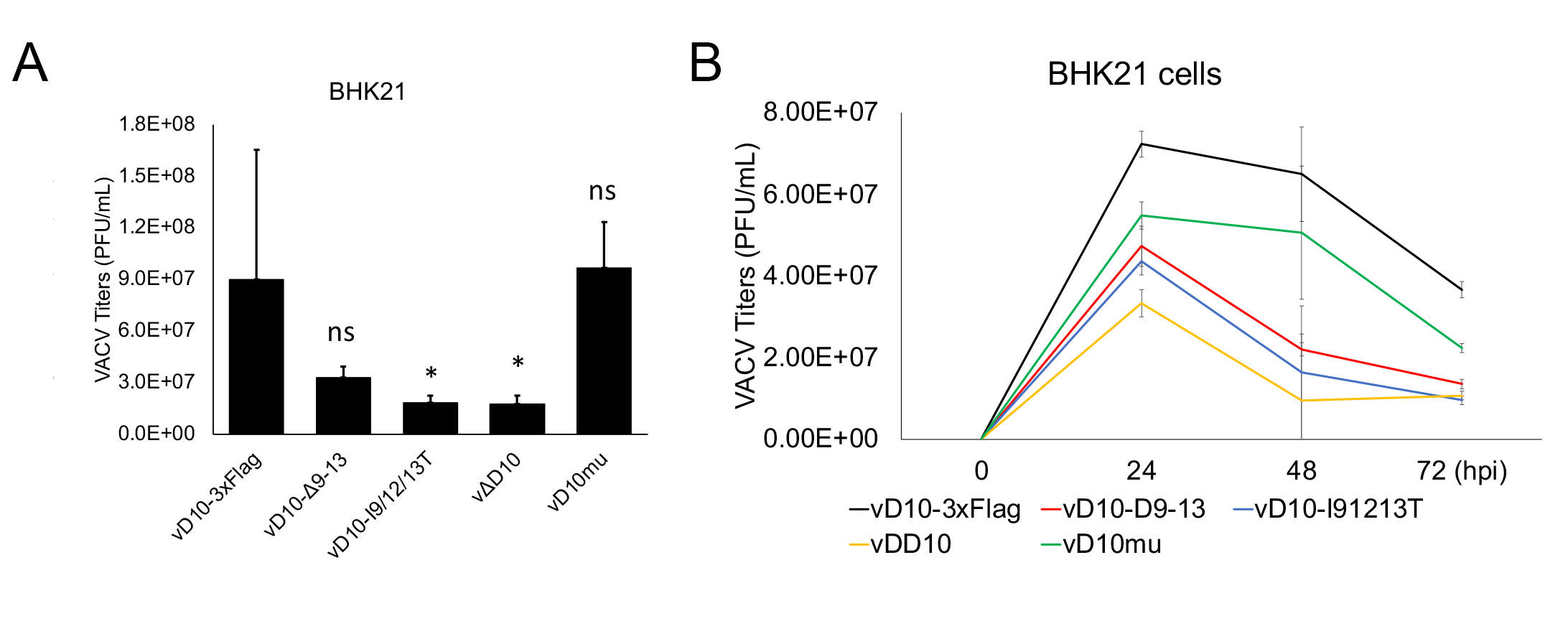

Supplement: FIG S6 [file mbio.00300-22-s0006.tif]
